# The Royal Army Medical Corps Training Manual

**Published:** 1908-12-26

**Authors:** 


### The Royal Army Medical Corps Section.

### THE ROYAL ARMY MEDICAL CORPS TRAINING MANUAL.

The publication of a new edition of the Royal Army Medical Corps Training Manual is a matter that calls for special notice, as its contents cannot but be regarded as a valuable indication of the view that the military authorities take of the scope and functions of an Army Medical Service. How much more comprehensive and enlightened that view is in the present time is well seen by a comparison of the manual of to-day with that of 1875, which was one of the first issued officially by the War Office and was designated a " Manual of Instructions for Non-Commissioned Officers and Men of the Army Hospital Corps." Its pages show that the work of the Medical Service of the Army was then limited to the treatment and transport of the sick and wounded soldiers, and there is no indication of that higher *rôle* of the prevention of disease which is now very properly regarded as the first and most important duty of an Army's Medical Service, and for which it should be specially organised. To get recognition of that principle has been a long and uphill fight, but it has been admitted, and the contents of this new Manual reflect how varied and wide-reaching have become the functions of the medical branch of the Army, and demonstrate clearly what an important part they may play in the conduct of war. For the officers of the Territorial Medical Service this new issue should have a special interest, as it is to be the text-book of instruction for all ranks of that corps. It cannot be called a reprint of its predecessor of 1904, though planned on the same lines.

The first part is devoted to the Training of the Royal Army Medical Corps in First Aid, Nursing, and the Prevention of Disease, a special chapter being added on Food and Cooking. All the first three subjects mentioned are fully and clearly dealt with. The nursing instruction is brought quite up to date, and the account of Antiseptics and Asepsis is in line with the views of the present day. The description of all the details connected with the conduct of an operation on modern lines is full, concise, and clear, and the whole chapter on Surgical Nursing leaves nothing to be desired. In view of the more enlightened position that has been assigned very properly to Sanitation as one of the most important principles underlying the conduct of all wars, we have turned with interest to this subject to ascertain how it has been dealt with, and we are pleased to find that it has received full and detailed consideration in a most excellent chapter on the Prevention of Disease. Clear and explicit in its treatment of this important branch of the Army surgeon's work, it is a very complete *résumé* of the knowledge that our different wars and the experience of peace training in camp and in manœuvres has brought us, and we notice with satisfaction that the character and scope of the instruction takes into consideration the different individualities that have to be not only instructed, but interested in this subject. We know that the lesson that has to be taught the nation and the soldier, both officer and man, is that the fighting power of an army in the field depends on its health, and that the latter in its turn hangs on the knowledge of sanitary matters the Army has acquired during peace training. Further, it has to be brought home to all ranks that no sanitary organisation will have any success in war save by the individual effort of every officer and man in the Army. Upon this point there is still a good deal of lingering prejudice amongst some of the older combatant officers, and there has not yet passed away the baneful effects of the doctrine taught by Lord Wolseley when Commander-in-Chief, and openly given expression to, that " the sanitary officer is the creation of recent years, and, as a rule, is a very useless functionary." Wiser counsels, however, have now prevailed, and we feel sure that if the advice of the present Army Medical Staff is followed, our Army, both Regular and Territorial, will be furnished with a reliable and efficient sanitary organisation that will stand the stress and test of war. Not only has there been created, as there should be, an excellent and responsible directing staff for laying down definitely all that is necessary as to the kind of instruction to be given and the procedure to be followed, but there is also provided a trained subordinate executive staff for carrying out orders. The pages of the new Manual show, too, that instruction is not limited to imparting a knowledge of general sanitation, but the importance of personal hygiene has not been overlooked. The Japanese war emphasised the latter fact, and the concluding pages of the chapter on Prevention of Disease are devoted to advice to the individual soldier on matters pertaining to the care of his body, to precautions while marching, and to the observance of care against infectious diseases. Emphasis is laid on the fact that of these latter typhoid fever and dysentery are essentially the scourges of an army in the field, and that attention to his food and drink are the best preventives against these two subtle and dangerous foes. Very properly special weight is attached to the necessity of never drinking non-officially authorised water, and of avoiding uncooked food. A special chapter has been devoted to general instructions as to the best and easiest methods of cooking.

The drills and exercises of the R.A.M.C. occupy the second part of the Manual, and it is satisfactory to note that the squad and company drill has been reduced to a minimum. It has at last been recognised that instruction in more than the elements of it are unnecessary and a waste of time. In the same way we hope the old ceremonial parades are to be a thing of the past. They were necessary in former years to earn the capitation grant, but the energies of all ranks were wasted in making a satisfactory appearance before the inspecting officer. Field training is fully and clearly described, and we feel certain that by the aid of this new edition of the R.A.M.C. Training Manual all ranks of the Medical Service of the Territorial Army should be able to make themselves proficient in their work and should find in its pages all the information they require.

